# Interventions, adaptive strategies and best practices improving maternal nutritional health in changing climatic conditions and diverse cultural contexts in low- and middle-income countries: a systematic review protocol

**DOI:** 10.1136/bmjopen-2025-107469

**Published:** 2025-12-18

**Authors:** S Bhanbhro, Corinna Thellmann, Zahid Ali Memon, Wardah Ahmed, Fizza Ansar, Saeed Ali, Shahbaz Ali, Munazza Khan, Hora Soltani

**Affiliations:** 1 Centre for Applied Health and Social Care Research (CARe), Sheffield Hallam University, Sheffield, UK; 2 Department of Community Health Sciences, The Aga Khan University, Karachi, Pakistan

**Keywords:** Nutrition, Maternal medicine, Climate Change, NUTRITION & DIETETICS, Systematic Review

## Abstract

**Abstract:**

**Background:**

The impact of poor nutritional health on maternal and infant morbidity and mortality remains high in low- and middle-income countries (LMICs), exacerbated by climate change-linked disasters. Maternal nutritional health, as a modifiable factor, is influenced by various social, political, economic and environmental factors, as well as cultural practices. Identifying climate change-related interventions, adaptive strategies and best practices targeting maternal nutritional health and well-being in LMICs within a cultural context helps inform the co-production of sustainable, culturally sensitive interventions to improve health outcomes for mothers and babies.

**Methods and analysis:**

We will undertake a systematic review of the literature employing the six steps of the Protocol, Search, Appraisal, Synthesis, Analysis and Report (PSALSAR) framework, including both peer-reviewed and grey literature. The Population, Concept and Context approach will be used to formulate the review question and the inclusion and exclusion criteria. We will include primary research comprising all study designs published in English from 2007 onwards. We will conduct searches in online academic databases, including CINAHL, MEDLINE, Global Health (CABI), AGRIS (FAO) and SCOPUS, as well as defined grey literature sources (ie, Google Scholar). Titles, abstracts and later full-text articles will be independently accessed and screened for eligibility criteria by four researchers. Following the integrative review methodology, we will present findings narratively, organised around the components of the PSALSAR framework to provide a comprehensive synthesis of the available evidence.

**Ethics and dissemination:**

As no primary data will be collected, the systematic review does not require formal ethics approval. However, we will give attention to ethical considerations within the identified studies. Findings will be published in a peer-reviewed journal and presented at relevant conferences. The findings of our systematic review and the ethnographic component of our research project will inform the development of appropriate maternal nutritional health interventions using co-production methodology in Pakistan.

**PROSPERO registration number:**

CRD420251080897.

**STRENGTHS AND LIMITATIONS OF THIS STUDY**

- We will follow the six steps of the Protocol, Search, Appraisal, Synthesis, Analysis and Report framework, which includes developing a protocol and search strategy, conducting appraisal, synthesis, analysis and reporting.
- We will assess the strength of the body of evidence using Grading of Recommendations Assessment, Development and Evaluation as the primary framework, but will adapt the criteria appropriately for each evidence type.
- Preferred Reporting Items for Systematic Reviews and Meta-Analyses 2020 guidelines will ensure transparency in methodology and rigour in the review process.
- We will be restricted in our search and focus on specific databases and grey sources, published in English and from 2007 onwards.

## Introduction

 Climate change is a significant global challenge which has led to an increase in extreme weather events. In many regions, temperature has risen, the water cycle has been disrupted, sea levels have gone up, and cyclones, heavy rainfall and flooding have increased.^1^ Women are the largest and most vulnerable group affected by climate-induced disasters.^2 3^ Pregnancy and childbirth, along with gender inequalities and socio-cultural norms, negatively affect women and girls, worsening these vulnerabilities.^4 5^

Climate hazards are particularly severe in low- and middle-income countries (LMICs), which are often the least able to respond to these climate ‘shocks’.^6^ Maternal and newborn health in LMICs is severely affected by climate change-linked disasters, mainly due to extreme heat,^7^ repeated flooding,^8 9^ drought and displacement following these disasters.^10^,^12^ Furthermore, pre-term birth (PTB) and low birth weight (LBW) remain the leading causes of neonatal and under-5 deaths.^13 14^ Both PTB and LBW, as well as maternal morbidity and mortality, can often be traced back to poor nutrition, a fundamental cause of anaemia.^15^,^17^ The impact of poor nutritional health status on maternal and infant morbidity and mortality remains high in LMICs,^15 16 18^ which is exacerbated by natural disasters.^19^,^21^

The nutrition of mothers before and during pregnancy thus plays a vital role in the short- and long-term health of a mother and her growing fetus.^22^,^24^ While maternal nutrition is a key determinant of mortality and morbidity for both mothers and infants, it is influenced by a variety of social, political, economic, and environmental factors and cultural practices. Research conducted in LMICs demonstrates the substantial impact of social and cultural context on maternal nutrition and health.^25^,^28^ Examples of cultural practices which could impact maternal nutrition and health in LMICs include taboos influencing women’s choices about what to eat and what not to eat during pregnancy,^28^,^36^ as well as post-delivery and while breastfeeding,^27 28 36 37^ culturally determined gender disparities in food distribution favouring male household members^25 38^ and maintaining household hierarchies.^35^ However, there exist beneficial cultural practices which could be channelled to improve maternal nutritional health status, such as ‘khairayat ge mani’ (charitable offering of food to neighbours/community at least once a day in the name of their deceased family members) or family-oriented pregnancy support found in Pakistan, which is not often explored within literature. As pregnancy and childbirth are distinct phases of life embedded in a cultural context, such beneficial practices can be systematically directed towards co-producing sustainable, culturally acceptable, community-based interventions that improve maternal nutrition and reduce health inequalities for mothers and their families.

Understanding the intersections between maternal nutritional health, cultural practices and climate-linked risks through the lens of wider social determinants requires a deep exploration of how different factors, processes, power constellations, institutions and interests affect maternal nutrition and health within households, communities, cultures and regions. Reviews have been conducted on the impact of climate change on maternal (nutritional) health,^79 12 19 21 39^,^41^ on the cultural impact on maternal nutrition,^2629 31 42^,^46^ on interventions for maternal nutritional health improvements,^47^,^52^ as well as on adaptation responses protecting maternal and child health from climate change impacts.^40^ However, the impact of climate change on maternal health and nutrition, and the utilisation of positive cultural practices to enhance maternal nutritional health, has not been investigated in LMICs.

### Aim and objectives

We aim to undertake a systematic review of literature exploring interventions, adaptive strategies and best practices within regional contexts identified as improving maternal nutritional health and well-being by mitigating the adverse effects of climatic conditions in LMICs. Specifically, we aim to

1. Identify climate change-related interventions, adaptive strategies and best practices targeting maternal nutritional health and well-being in LMICs.
2. Examine the role of cultural practices in facilitating and hindering maternal nutritional health in these settings.
3. Synthesise evidence on effectiveness, gaps and contextual factors influencing outcomes.
4. Identify facilitators and adaptation strategies implemented by women and their communities, healthcare providers, and non-governmental and governmental institutions/organisations (relevant parties) to mitigate the impact of climate change on maternal nutritional health in LMICs.
5. Identify culturally accepted interventions and programmes designed to improve climate-related nutritional challenges experienced by pregnant and breastfeeding women in LMICs.

## Methods and analysis

We will undertake a systematic review of the literature using the six steps of the Protocol, Search, Appraisal, Synthesis, Analysis and Report (PSALSAR) framework,^53^ which includes developing a protocol and search strategy, conducting appraisal, synthesis, analysis and reporting. The systematic review protocol is guided by the criteria set out in the Preferred Reporting Items for Systematic Review and Meta-Analysis Protocols (PRISMA-P) checklist.^54^ If need be, we will document important protocol amendments in PROSPERO. We will use Covidence, a web-based programme, to manage and streamline our systematic review.

The review aims to include studies with diverse methods, participants and outcomes (table 2). Consequently, instead of a meta-analysis, we will use an integrative review of the empirical literature approach^55 56^ and present the review results narratively.

### Review question framework

The Population, Concept and Context (PCC) framework^57^ was used to formulate the review question, examining which interventions, adaptive strategies and best practices enable women’s maternal nutritional health and well-being in the context of changing climatic conditions and cultural perspectives in LMICs.

### Databases and search strategy

We will include the online databases CINAHL, MEDLINE, Global Health (CABI), AGRIS (FAO) and SCOPUS, as well as grey sources, namely Google Scholar, WHO publications, UNICEF reports, FAO documents, World Bank reports, IOM reports, reports from regional nutrition initiatives (eg, SAARC Food Bank), NGO programme evaluations and government policy documents and programme reports.

For our systematic review, we will employ search terms and strategies summarised in table 1 (see online supplemental appendix for an example of a CINAHL search). We will focus on pregnant and/or breastfeeding women in LMICs and the impact of climate change and cultural context on their nutrition. Given the significance of these aspects mentioned, we will seek interventions, adaptive strategies and best practices. We will include primary research, including qualitative and quantitative research and mixed methods designs, comprising all study designs. Given the scope of the systematic review, we will conduct hand searches of references, review key organisational reports from the WHO, UNICEF and WFP, and consult regional databases such as African Journals Online (AJOL) and SciELO.

**Table 1:** Keywords and possible search combinations using Boolean (OR, AND, NOT) strings

Search line	Concept	Search terms
S1	Population	(maternal OR “pregnant women” OR pregnancy OR “breastfeeding women” OR lactation OR “lactating women” OR nursing OR “nursing women” OR prenatal OR antenatal OR perinatal OR postpartum)
S2	Nutrition	(nutrition OR “nutritional status” OR diet OR “dietary diversity” OR “food security” OR “food insecurity” OR “food access” OR malnutrition OR “undernutrition” OR “micronutrient deficiency” OR anaemia OR “maternal health” OR “dietary intake”)
S3	Interventions	(intervention* OR program* OR programme* OR strategy OR strategies OR “best practice*” OR “adaptive strateg*” OR “adaptation strateg*” OR “community based” OR “behaviour change” OR “behaviour change” OR implementation OR “nutrition education” OR supplement* OR “food assistance” OR “nutritional support”)
S4	Climate change	(“climate change” OR “climate variability” OR “climate adaptation” OR “extreme weather” OR disaster OR heat* OR drought OR flood* OR “environmental change” OR “food availability” OR “climate resilience” OR “climate vulnerability” OR “climate impact” OR “climate risk”)
S5	Cultural context	(cultur* OR “cultural context” OR “cultural practice” OR “cultural factors” OR “cultural taboo” OR “food beliefs” OR “dietary beliefs” OR “sociocultural factors” OR “religious practices” OR “gender norms” OR “traditional practices” OR “social factors” OR “cultural sensitivity” OR “cultural adaptation”)
S6	Study design	(RCT OR “randomized controlled trial” OR “randomised controlled trial” OR “quasi-experimental” OR “pre-post” OR cohort OR “case control” OR “cross-sectional” OR “mixed methods” OR qualitative OR “implementation research” OR “program evaluation” OR “intervention study” OR “effectiveness study”)
S7	Geographic	ScHARR LMIC Filter^64^ (see detailed filter in online supplemental appendix)
S8	**Final combination**	**S1 AND S2 AND S3 AND (S4 OR S5) AND S6 AND S7**

LMIC, low- and middle-income country; ScHARR, School of Health and Related Research.

### Eligibility

The PCC framework^57^ will be used to determine articles’ eligibility and exclusion from selection; see table 2. We will include studies and grey literature published between 2007 and the present. This timeframe captures growing global attention to climate change (such as the release of landmark reports by the Intergovernmental Panel on Climate Change in 2007^58^ and the 2008 World Health Assembly resolutions,^59 60^ nutrition, major climate events in Pakistan (eg, major floods in 2010, 2022), and nearly two decades of relevant research, policy and programme developments.

**Table 2:** Inclusion and exclusion criteria

	Inclusion criteria	Exclusion criteria
Population	Pregnant women and breastfeeding mothers Studies focusing on families, communities or healthcare providers will be included if they address maternal nutrition for the specified population	Girls under reproductive age Studies focusing on families, communities or healthcare providers that do not address maternal nutrition for the specified population
Concept(s)	Maternal nutritional health status, practices and outcomes Cultural influences on maternal nutrition Climate-related impacts on maternal nutrition (extreme weather events, seasonal variations, climate-related disease outbreaks) Intersection between cultural contexts and climate-related events affecting maternal nutrition Interventions to improve maternal nutritional health Women’s and communities’ adaptation strategies and knowledge related to maternal nutrition Cultural acceptability and appropriateness of nutritional interventions	Studies that do not focus on the intersection of maternal nutritional health, climate change and cultural contexts Studies that do not suggest or investigate interventions, adaptation strategies or best practices aiming at improving maternal nutritional health, considering climate change and cultural context
Context	LMICs as defined by the School of Health and Related Research (ScHARR) LMIC filter^64^ Rural, urban, semi-urban, coastal and wetland settings Areas experiencing climate-related events (floods, droughts, heat waves, etc) Various healthcare settings (community, primary care, hospital) Cultural institutional contexts Household and community contexts	Studies from HICs
Study types	Peer-reviewed published articles Grey literature, as highlighted under grey sources, is considered	Studies that lack well-defined theoretical approaches and/or methodology (eg, reports, essays, news articles, commentary, etc) Grey literature not mentioned under grey sources is not considered
Year	Studies and grey literature published between 2007 and the present	Studies and grey literature published before 2007
Language	English	Any other language besides English

HICs, high-income countries; LMICs, low- and middle-income countries.

### Data collection and analysis

We will upload and review titles and abstracts from database searches and the sources in Covidence and remove duplicates. Titles, abstracts and later full-text articles will be screened independently by four researchers (CT, FA, WA and ShA) for eligibility criteria. Any study that does not meet the inclusion criteria during the review will be removed. We will keep a log of excluded studies, stating the reason for exclusion. We will use the PRISMA-P flowchart to summarise the screening and selection steps for the article.

Four authors (CT, SB, SaA and MK) will independently assess the quality of the included literature using the Joanna Briggs Institute (JBI) Critical Appraisal Checklists^61^ adapted to each study design, noting how researchers addressed potential biases. The assessment of risk of bias will inform the interpretation of the review findings. Studies identified as high-risk will not be excluded from the review; however, their influence on the overall conclusions will be examined through sensitivity analyses. Where appropriate, subgroup or sensitivity analyses will be conducted to explore the potential impact of studies with a higher risk of bias on the robustness of the review findings. The results of the risk of bias assessments will be presented in tabular form and discussed narratively to provide a transparent account of study quality across the included evidence. We will assess the strength of the body of evidence using the Grading of Recommendations Assessment, Development and Evaluation^62^ approach as the primary framework, but will adapt the criteria appropriately for each evidence type. We will not assess meta-bias as we will be doing an integrative review.^55 56^ Preferred Reporting Items for Systematic Reviews and Meta-Analyses (PRISMA) 2020 guidelines will further ensure transparency in methodology and rigour in the review process.^63^

In cases of disagreement and ambiguity, we will deliberate on inclusion with a third reviewer (HS and/or ZM).

### Data extraction and synthesis

According to PRISMA guidelines,^63^ a flowchart will be created to illustrate each stage of the selection process, including screening at the title, abstract and full-text levels. A standardised form developed a priori will be used to capture study characteristics (eg, design, methods, including data collection and analysis, setting and sample size), participant characteristics, primary and secondary outcomes (see box 1), main study findings and stated limitations. Following the integrative review methodology,^55 56^ we will present findings narratively, organised around the components of the PSALSAR framework^53^ to provide a comprehensive understanding of the evidence. Using the PCC framework, the data from all included studies will be sought for the items described in table 3.

Box 1 **Data extraction items**

**Primary outcomes**

- Evidence of improved maternal nutritional health (eg, dietary intake, micronutrient status, nutritional deficiencies) resulting from interventions, adaptive strategies or best practices in LMICs.
- Reduction in climate-related nutritional risks for pregnant and breastfeeding women (eg, food insecurity, undernutrition, micronutrient deficiencies) linked to identified strategies.

**Secondary outcomes**

- Influence of cultural practices (both enabling and restrictive) on the success or failure of interventions.
- Identification of contextual factors (socioeconomic, gender, health system, community structures) shaping intervention outcomes.
- Evidence on the acceptability, feasibility and sustainability of interventions in diverse cultural settings.
- Documentation of community, institutional and policy-level adaptation efforts addressing maternal nutritional health in the context of climate change.

LMICs, low- and middle-income countries.

**Table 3:** 

Population	Concept	Context
Description of the population (pregnant women and lactating women) Sociodemographic characteristics (age, ethnicity, education, income, etc) Health status indicators (nutritional status, pre-existing health conditions) Vulnerable subgroups (eg, rural populations, indigenous communities, displaced populations)	Type of intervention, strategy or practice (nutrition-specific or nutrition-sensitive) Description of intervention (content, duration, frequency, delivery method) Adaptive strategies related to climate change (eg, livelihood diversification, food security measures) Best practices reported (community engagement, culturally tailored approaches) Outcomes measured (maternal nutritional status, dietary diversity, food security, health behaviours) Reported effectiveness (qualitative or quantitative) Barriers and facilitators to implementation Theoretical or conceptual frameworks applied (if any)	Country or region (classified as LMIC per World Bank) Cultural context (social norms, gender roles, traditional practices) Climate-related factors (climate risks, environmental challenges, food system disruptions) Setting (rural, urban, peri-urban, coastal, wetlands) Policy or health system context (availability of health and nutrition services) Timeframe of intervention or study

LMIC, low- and middle-income country.

### Ethics and dissemination

This systematic review is part of a study titled, ‘Birthing at burning places: an ethnographic study of intersections among climate-linked risks, maternal nutritional health, and cultural practices in Sindh, Pakistan’, and we have obtained ethics approval for the overall project from Sheffield Hallam University (ER79599950), Aga Khan University Research Ethics Committees (Ref: 2025-11063-34591) and National Bio-ethics Committee for Research (Ref. No. 4-87/NBCR-1273/24-25/379).

As no primary data will be collected, the systematic review does not require formal ethics approval. However, we will give attention to ethical considerations within the identified studies. Findings will be published in a peer-reviewed journal and presented at relevant conferences. The findings of our systematic literature review will help inform the ethnographic context of our broader research project, which explores the intersections among climate-linked risks, maternal nutritional health and cultural practices in Pakistan. Finally, the findings of this review and the ethnographic component of our research project will inform the co-production of community-based culturally sensitive interventions to tackle growing climate-related maternal nutritional health risks in Pakistan.

### Patient and public involvement

In the development of this protocol, there was no patient and public involvement.

## Discussion

Climate-related events are increasing, exacerbating the poor nutritional health of mothers and infants in LMICs. Cultural contexts inform maternal and infant nutritional health and responses to climate-related events. While other reviews focusing on LMICs have examined the impact of climate-related events on maternal (nutritional) health^79 12 19 21 39^,^41^ and the impact of cultural contexts on maternal nutritional health,^26 29 31 42^ to date, there exists a gap in literature on the intersection of the impact of both climate-related events and cultural contexts on maternal nutritional health in LMICs. Furthermore, studies on interventions and best practices to address and improve the intersection of climate-related events, cultural context and maternal nutritional health have been lacking. By focusing on both the intersection of climate-related events, cultural contexts and maternal nutritional health as well as on interventions and best practices responding to this intersection in LMICs, our systematic review will contribute to several Sustainable Development Goals (SDGs), namely SDG 2 (Zero Hunger), SDG 3 (Good Health and Well-being), SDG 5 (Gender Equality) and SDG 13 (Climate Action). Based on the findings of our systematic review, we will propose recommendations for future research directions and priorities to address the identified gaps and enhance understanding of the intersection of maternal nutritional health challenges, climate-related events and cultural contexts in LMICs. By uncovering how climate-related events and cultural contexts might influence the success of maternal nutritional health interventions in LMICs, we will provide valuable insights for co-development and implementation evaluation of maternal nutritional health interventions to enhance outcomes for mothers and babies, particularly in regions with the highest incidence of maternal and neonatal mortality and morbidity.

We will be restricted in our search and focus on specific databases and grey sources. This might prevent us from capturing relevant studies published in other databases and grey sources. Furthermore, we might miss relevant studies published in languages other than English and before 2007, our cut-off point. Given the large number of LMICs, we might miss some countries, not only because of filter limitations but also due to language restrictions, limited accessibility to country-specific grey literature and gaps in international databases. Additionally, the heterogeneity of study designs might complicate our data synthesis and analysis, and the transferability of findings might be limited due to diverse contexts.

## Supplementary material

10.1136/bmjopen-2025-107469 online supplemental file 1

10.1136/bmjopen-2025-107469 online supplemental file 2
