# Supplementary material for: Interventions, adaptive strategies and best practices improving maternal nutritional health in changing climatic conditions and diverse cultural contexts in low- and middle-income countries: a systematic review protocol
Source: BMJ Open. 2025 Dec 18;15(12):e107469. doi: 10.1136/bmjopen-2025-107469 (PMC12716598; doi:10.1136/bmjopen-2025-107469)
Supplement: online supplemental file 2 [file bmjopen-15-12-s002.docx]

**APPENDIX: Search Strategy for CINAHL**

**CINAHL via EBSCO Search Date:** July 2, 2025

**Search Mode**: Proximity

**Years:** 2007-2025

**Language:** English

**Expanders:** Apply equivalent subjects; Also search within full text of articles

**Search Strategy:**

1. (maternal OR "pregnant women" OR pregnancy OR "breastfeeding women" OR lactation OR "lactating women" OR nursing OR "nursing women" OR prenatal OR antenatal OR perinatal OR postpartum)

2. (nutrition OR "nutritional status" OR diet OR "dietary diversity" OR "food security" OR "food insecurity" OR "food access" OR malnutrition OR "undernutrition" OR "micronutrient deficiency" OR anaemia OR "maternal health" OR "dietary intake")

3. (intervention* OR program* OR programme* OR strategy OR strategies OR "best practice*" OR "adaptive strateg*" OR "adaptation strateg*" OR "community based" OR "behaviour change" OR "behavior change" OR implementation OR "nutrition education" OR supplement* OR "food assistance" OR "nutritional support")

4. ("climate change" OR "climate variability" OR "climate adaptation" OR "extreme weather" OR disaster OR heat* OR drought OR flood* OR "environmental change" OR "food availability" OR "climate resilience" OR "climate vulnerability" OR "climate impact" OR "climate risk")

5. (cultur* OR "cultural context" OR "cultural practice" OR "cultural factors" OR "cultural taboo" OR "food beliefs" OR "dietary beliefs" OR "sociocultural factors" OR "religious practices" OR "gender norms" OR "traditional practices" OR "social factors" OR "cultural sensitivity" OR "cultural adaptation")

6. (RCT OR "randomized controlled trial" OR "randomised controlled trial" OR "quasi-experimental" OR "pre-post" OR cohort OR "case control" OR "cross-sectional" OR "mixed methods" OR qualitative OR "implementation research" OR "program evaluation" OR "intervention study" OR "effectiveness study")

7. ((MH "Developing Countries") OR (Africa or Asia or Caribbean or West Indies or South America or Latin America or Central America) OR (Afghanistan or Albania or Algeria or Angola or Antigua or Barbuda or Argentina or Armenia or Armenian or Aruba or Azerbaijan or Bahrain or Bangladesh or Barbados or Benin or Byelarus or Byelorussian or Belarus or Belorussian or Belorussia or Belize or Bhutan or Bolivia or Bosnia or Herzegovina or Hercegovina or Botswana or Brasil or Brazil or Bulgaria or Burkina Faso or Burkina Fasso or Upper Volta or Burundi or Urundi or Cambodia or Khmer Republic or Kampuchea or Cameroon or Cameroons or Cameron or Camerons or Cape Verde or Central African Republic or Chad or Chile or China or Colombia or Comoros or Comoro Islands or Comores or Mayotte or Congo or Zaire or Costa Rica or Cote d'Ivoire or Ivory Coast or Croatia or Cuba or Cyprus or Czechoslovakia or Czech Republic or Slovakia or Slovak Republic or Djibouti or French Somaliland or Dominica or Dominican Republic or East Timor or East Timur or Timor Leste or Ecuador or Egypt or United Arab Republic or El Salvador or Eritrea or Estonia or Ethiopia or Fiji or Gabon or Gabonese Republic or Gambia or Gaza or Georgia Republic or Georgian Republic or Ghana or Gold Coast or Greece or Grenada or Guatemala or Guinea or Guam or Guiana or Guyana or Haiti or Honduras or Hungary or India or Maldives or Indonesia or Iran or Iraq or Isle of Man or Jamaica or Jordan or Kazakhstan or Kazakh or Kenya or Kiribati or Korea or Kosovo or Kyrgyzstan or Kirghizia or Kyrgyz Republic or Kirghiz or Kirgizstan or Lao PDR or Laos or Latvia or Lebanon or Lesotho or Basutoland or Liberia or Libya or Lithuania or Macedonia or Madagascar or Malagasy Republic or Malaysia or Malaya or Malay or Sabah or Sarawak or Malawi or Nyasaland or Mali or Malta or Marshall Islands or Mauritania or Mauritius or Agalega Islands or Mexico or Micronesia or Middle East or Moldova or Moldovia or Moldovian or Mongolia or Montenegro or Morocco or Ifni or Mozambique or Myanmar or Myanma or Burma or Namibia or Nepal or Netherlands Antilles or New Caledonia or Nicaragua or Niger or Nigeria or Northern Mariana Islands or Oman or Muscat or Pakistan or Palau or Palestine or Panama or Paraguay or Peru or Philippines or Philipines or Phillipines or Phillippines or Poland or Portugal or Puerto Rico or Romania or Rumania or Roumania or Russia or Russian or Rwanda or Ruanda or Saint Kitts or St Kitts or Nevis or Saint Lucia or St Lucia or Saint Vincent or St Vincent or Grenadines or Samoa or Samoan Islands or Navigator Island or Navigator Islands or Sao Tome or Saudi Arabia or Senegal or Serbia or Montenegro or Seychelles or Sierra Leone or Slovenia or Sri Lanka or Ceylon or Solomon Islands or Somalia or South Africa or Sudan or Suriname or Surinam or Swaziland or Syria or Tajikistan or Tadzhikistan or Tadjikistan or Tadzhik or Tanzania or Thailand or Togo or Togolese Republic or Tonga or Trinidad or Tobago or Tunisia or Turkey or Turkmenistan or Turkmen or Uganda or Ukraine or Uruguay or USSR or Soviet Union or Union of Soviet Socialist Republics or Uzbekistan or Uzbek or Vanuatu or New Hebrides or Venezuela or Vietnam or Viet Nam or West Bank or Yemen or Yugoslavia or Zambia or Zimbabwe or Rhodesia) OR ((developing or less* developed or under developed or underdeveloped or middle income or low* income or underserved or under served or deprived or poor*) N1 (countr* or nation? or population? or world)) OR ((developing or less* developed or under developed or underdeveloped or middle income or low* income) N1 (economy or economies)) OR (low* N1 (gdp or gnp or gross domestic or gross national)) OR (low N3 middle N3 countr*) OR (lmic or lmics or third world or lami countr*) OR transitional countr* OR ((high burden or high-burden or countdown) N1 countr*))
