# Supplementary material for: Interventions, adaptive strategies and best practices improving maternal nutritional health in changing climatic conditions and diverse cultural contexts in low- and middle-income countries: a systematic review protocol
Source: BMJ Open. 2025 Dec 18;15(12):e107469. doi: 10.1136/bmjopen-2025-107469 (PMC12716598; doi:10.1136/bmjopen-2025-107469)
Supplement: Supplementary file 3 [file bmjopen-2025-107469.reviewer_comments.pdf]

PEER REVIEW HISTORY

BMJ Open publishes all reviews undertaken for accepted manuscripts. Reviewers are asked to complete a checklist review form and are provided with free text boxes to elaborate on their assessment. These free text comments are reproduced below.

ARTICLE DETAILS

Title (Provisional)

Interventions, adaptive strategies, and best practices improving maternal nutritional health in changing climatic conditions and diverse cultural contexts in low- and middle-income countries: A Systematic Review Protocol

Authors

Bhanbhro, S; Thellmann , Corinna; Memon, Zahid Ali; Ahmed, Wardah; Ansar, Fizza; Ali, Saeed; Ali, Shahbaz; Khan, Munazza; Soltani, Hora

---

VERSION 1 - REVIEW

**Reviewer**	**1**
**Name**	**Bhattarai, Nawaraj**
**Affiliation**	**Institute of Health & Society, Newcastle University**
**Date**	**09-Nov-2025**
**COI**	**None**

This is a well written protocol and as a protocol there are no major concerns. However there are few minor issues, I would like the authors to look at:

1. Considering the differences in the cultural contexts across the LMICs, the interventions, adaptive strategies and best practices that are effective in one country may not easily be implemented or promoted in another. I anticipate this issue will be addressed by the authors in their final project manuscript.

2. Is there a possibility of missing some LMICs due to potential unknown limitations in the search strategy to be used?

3. What is the rationale for not including the literature published prior to 2007 in the search strategy?

4. Considering this is a protocol, should not the "Patient and Public Involvement" section be written in future tense?

5. Could you please check the completeness of the following references : 3, 14. 53, 57, 59?

VERSION 1 - AUTHOR RESPONSE

Reviewer 1

Nr	Comment	Response	Location in Revised Manuscript
1	Considering the differences in the cultural contexts across the LMICs, the interventions, adaptive strategies and best practices that are effective in one country may not easily be implemented or promoted in another. I anticipate this issue will be addressed by the authors in their final project manuscript.	We very much appreciate this important observation and agree that culture and context play an important role in intervention, best practice, and adaptive strategy success. We anticipate this to be one of our systematic review's major findings, calling for the relevance of contextually grounded ethnographic research and the co-production of interventions.	
2	Is there a possibility of missing some LMICs due to potential unknown limitations in the search strategy to be used?	Thank you for raising this important concern. By employing the ScHARR LMIC filter, we are using an improved adaptation of the Cochrane EPOC LMIC geographic search filter for our search strategy, hoping this will improve inclusion of studies focusing on LMICs. However, both filters have not been validated yet. There is a possibility of missing some LMICs, not only because of filter limitations but language restrictions, limited accessibility of country-specific grey literature, and international database gaps. We have included this limitation in the discussion section.	Lines 244-251, highlighted in yellow

3	What is the rationale for not including the literature published prior to 2007 in the search strategy?	Thank you for raising this valuable question. We decided to include studies and grey literature published from 2007 onwards, as this timeframe captures growing global attention to climate change (such as the release of landmark reports by the Intergovernmental Panel on Climate Change (IPCC) in 2007 and the 2008 World Health Assembly resolutions), nutrition, major climate events in Pakistan (e.g., major floods in 2010, 2022), and nearly two decades of relevant research, policy, and programmatic developments.	We have mentioned this rationale in the original manuscript in lines 158-164. This rational can now be found in lines 151-156 of the revised manuscript, highlighted in yellow.
4	Considering this is a protocol, should not the "Patient and Public Involvement" section be written in the future tense?	We thank you for highlighting this. We have not involved PPI in our protocol development, but we will involve them in the actual review. We adjusted the phrasing in the revised manuscript.	Line 218, highlighted in yellow
5	Could you please check the completeness of the following references: 3, 14. 53, 57, 59?	Thank you very much for spotting this, we have adjusted the references.	As we have added new references to the revised manuscript, some of the corresponding numbers have changed:<br><br>Original reference 53 is now 56.<br><br>Original reference 57

			is now 60.<br><br>Original reference 59 is now 62.<br><br>We highlighted the references in yellow in the reference list.
